# Unusual Presentation of Papillary Thyroid Carcinoma as a Lateral Neck Cystic Mass: A Case Report

**DOI:** 10.7759/cureus.75863

**Published:** 2024-12-17

**Authors:** Abdulaziz S Altwijri, Ziyad K Aldhubayb, Abdulaziz S Al-lihimy

**Affiliations:** 1 Breast and Endocrine Surgery, King Fahad Specialist Hospital, Buraydah, SAU; 2 Breast and Endocrine Surgery, King Khalid University Hospital, Riyadh, SAU; 3 General Surgery, King Fahad Specialist Hospital, Buraydah, SAU

**Keywords:** case report, cystic mass, lateral neck, metastatic, papillary thyroid cancer

## Abstract

Papillary thyroid cancer (PTC) is the most frequent thyroid malignancy. Recently, the incidence has become widespread among both male and female individuals worldwide. In this article, we aim to report a 32-year-old Saudi female who presented with a painless lateral neck mass for more than seven months, and on excisional biopsy, was found to have features of PTC. Therefore, the patient underwent total thyroidectomy and central neck dissection with left lateral neck dissection with uneventful recovery, and final histopathology resulted in PTC with two out of thirteen central lymph nodes being positive for metastasis. PTC should always be included in the differential diagnosis when patients present with lateral neck cystic mass. Therefore, an excisional biopsy should be considered even if the fine-needle aspiration (FNA) result was benign.

## Introduction

Among thyroid malignancies, papillary thyroid cancer (PTC) is the most common [1]. The incidence has recently increased among both male and female individuals worldwide [2]. This is due to the increased number of well-established risk factors such as radiation exposure, obesity, diet, hormones, and family history [2]. Consequently, the most frequent presentation is thyroid nodules, with prevalence ranging from 19% to 67% among adult patients, and clinically the majority of them are silent [3,4].

PTC is considered an innocuous tumor. Nevertheless, most of the PTC cases are well-differentiated with low invasion and recurrence rates [1]. However, some of its variants, such as the diffuse sclerosing variant of papillary thyroid carcinoma, are more aggressive with distinct clinical presentations and pathological characteristics [5,6]. This article reports an unusual presentation of papillary thyroid carcinoma as a lateral cystic neck mass. 

## Case presentation

A 32-year-old Saudi female unknown to have any chronic illness, with no previous surgeries, was referred from another hospital as a case of left lateral neck mass. The patient complained of a painless neck mass for more than seven months that is stable in size and not increasing with time. She has no symptoms of hyperthyroidism or hypothyroidism. Also, the patient denies any history of dysphagia, sleep apnea, voice change, or other compressive symptoms. Also, history was unremarkable for similar complaints or ionizing radiation exposure. On the other hand, family history was positive for thyroid malignancy.

On examination, there was a palpable firm mass in the left lateral part of the neck, beneath the left sternocleidomastoid, oval in shape, with regular borders, a smooth surface, not fixed to the skin, and not moving with tongue protrusion or swallowing. Thyroid and cervical lymph nodes were not palpable.

**Figure 1:**
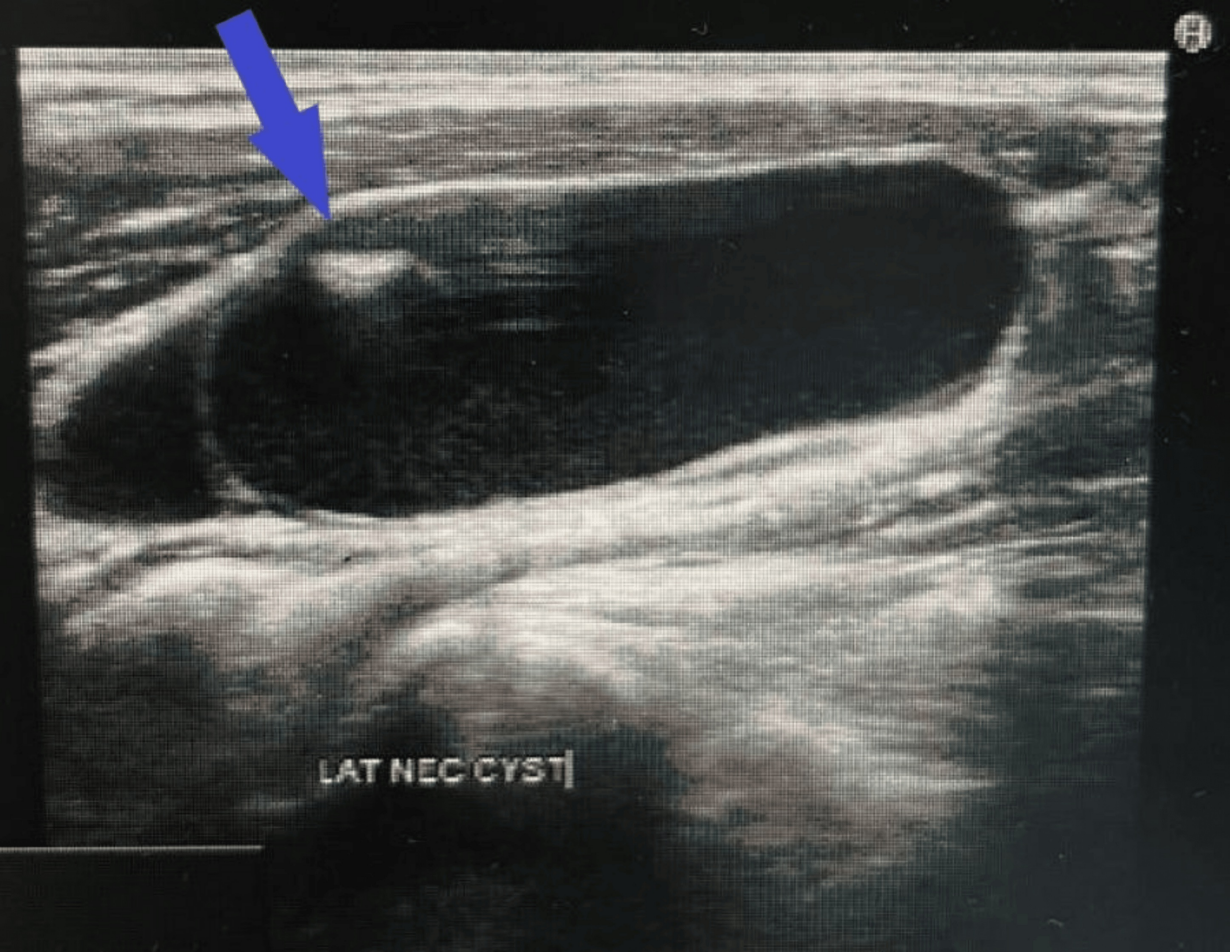
Ultrasound of the neck A 5.2 x 1.9 cm thick-walled lobulated cystic lesion in the left lateral neck, inferior to the left sternocleidomastoid muscle, with slight mass effect on the adjacent left jugular vein. It demonstrates internal solid components (blue arrow), debris, and a few septations.

**Figure 2:**
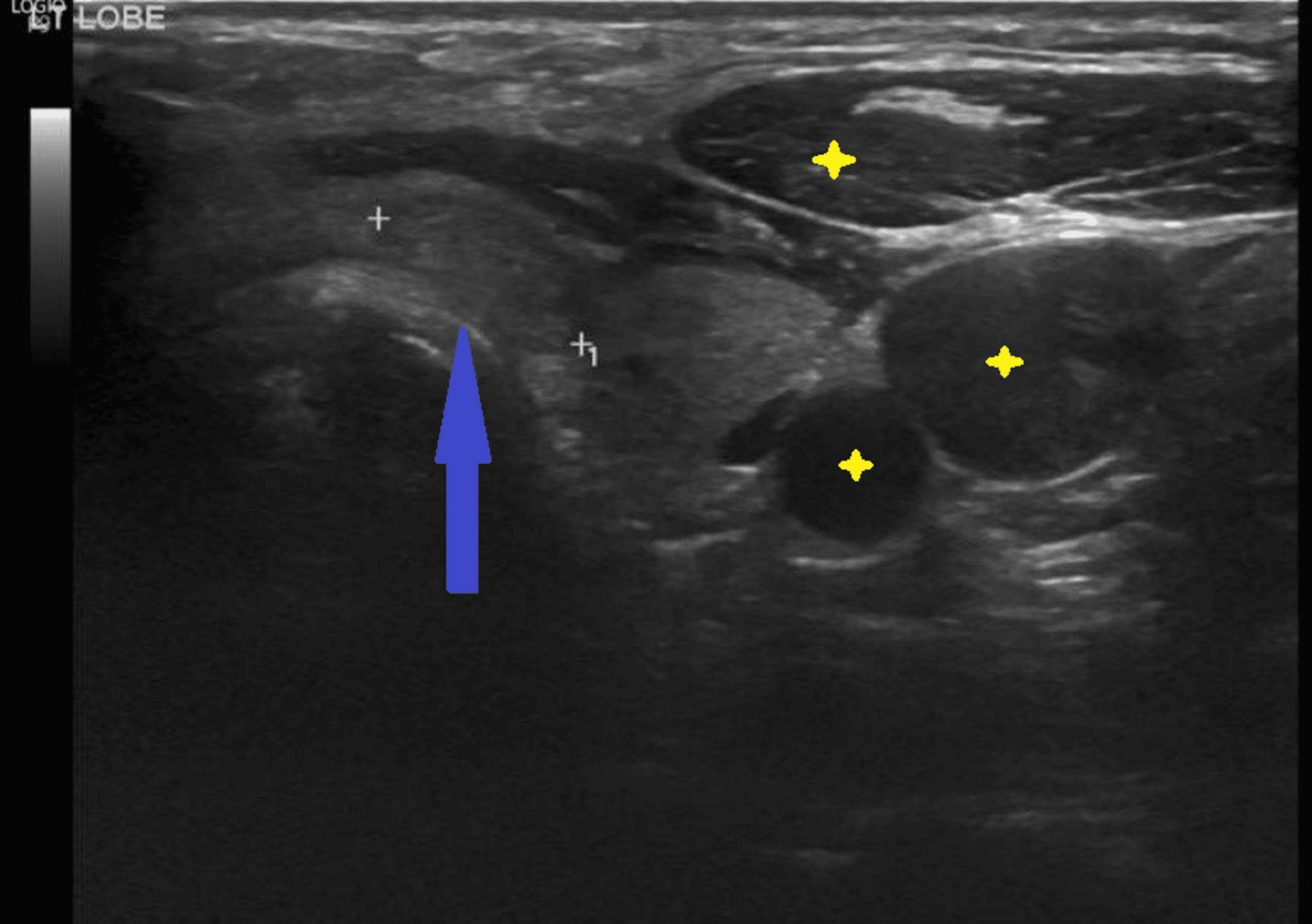
Uultrasound of the thyroid There are few small left thyroid lobe colloid cysts. The largest measured 7 mm (labelled by yellow stars), and the small, well-defined, solid isoechoic left isthmic junction measured 11x4 mm with no calcifications (pointed by a blue arrow). Thyroid Imaging Reporting and Data Systems (TIRADS)-3.

**Figure 3:**
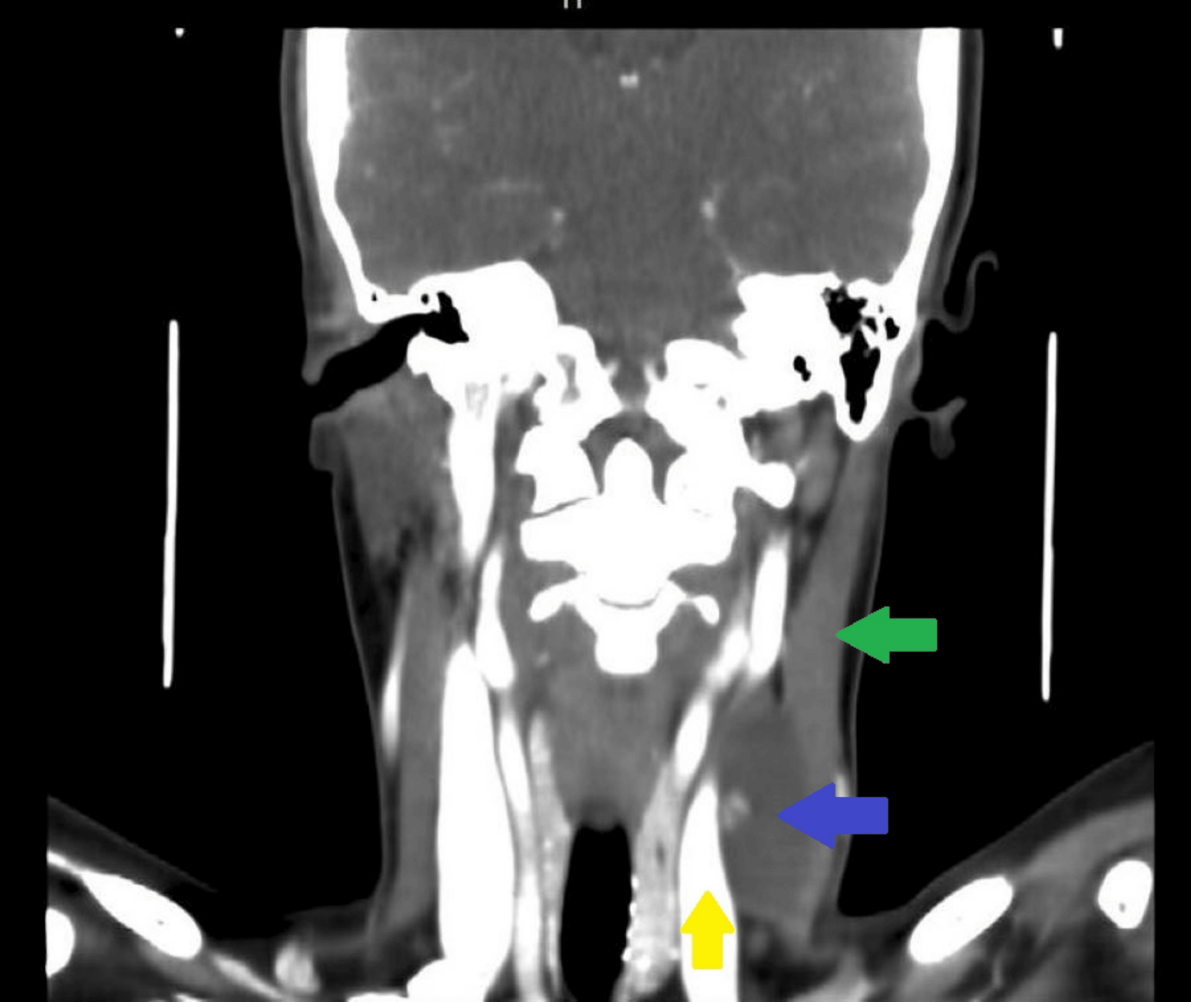
Computed tomography of the neck The sagittal view showed a well-defined left deep cervical cystic lesion with solid components measuring about 1.7x3x4.7 cm (blue arrow). The lesion exhibits a deep insinuation between the left sternocleidomastoid (green arrow) and jugular vein (yellow arrow).

Ultrasound (US) of the neck showed a 5.2x1.9 cm thick-walled lobulated cystic lesion in the left lateral neck, inferior to the left sternocleidomastoid muscle, with a slight mass effect on the adjacent left jugular vein. It demonstrates internal solid components, debris, and a few septations (Figure 1). There are a few small left thyroid lobe colloid cysts, the largest measuring 7 mm, and a small, well-defined, solid isoechoic left isthmic junction measuring 11x4 mm with no calcifications. There are few non-specific cervical lymphadenopathy bilaterally (Figure 2). In addition, a computed tomography (CT) scan of the neck reported a well-defined left deep cervical cystic lesion measuring about 1.7x3x4.7 cm. The lesion exhibits a deep insinuation between the left sternocleidomastoid and jugular vein (Figure 3). The patients who underwent excisional biopsy were deep to the sternocleidomastoid muscle and abutting the jugular vein. In consequence, histopathology sections show cystic and solid lesions with cysts lined by follicular epithelium and filled with colloids. Solid areas show papillary architecture and nuclear features of PTC. Therefore, the patient underwent total thyroidectomy and central neck dissection with left lateral neck dissection. Postoperatively, uneventful final histopathology resulted in 0.4 cm PTC, classic subtype, with two out of thirteen central lymph nodes being positive for metastasis PTC while sixteen left lateral lymph nodes were negative.

## Discussion

The most common presentation of thyroid cancer is an asymptomatic thyroid mass or a nodule [7]. Rarely, PTC presents as a lateral cystic neck mass without a palpable lesion in the thyroid gland [8,9]. Hence, most commonly lateral cervical cysts are benign, such as branchial cleft cysts. 

Jack M. Monchik et al. reported the incidence of PTC was 5.8% in patients presented with a cystic neck mass and no palpable thyroid lesion [10]. Furthermore, in the literature review, PTC presented as lateral neck cyst metastases from occult primary thyroid carcinoma in the range of 10-11% of cases [11,12].

Clinically, patients with PTC may present with lateral neck cystic swelling as an initial presentation, especially when the thyroid is unremarkable, mimicking benign cervical cysts [13]. Consequently, ultrasonography is an important diagnostic modality to assess thyroid nodules in addition to fine needle aspiration (FNA). Nevertheless, because of the nonexpert ultrasound operator and small thyroid nodules with benign features, these factors contributing to misdiagnosis on ultrasound may present. Therefore, excisional biopsy is necessary to detect metastatic PTC with negative FNA or very small thyroid lesions not amenable.

Finally, as the incidence of PTC increases, atypical presentation as a lateral neck cystic lesion should raise suspicion of PTC, also differentiated with more aggressive forms, such as anaplastic carcinoma and angiosarcoma, which present with clinical symptoms such as hoarseness or dysphagia [14]. Accordingly, excisional biopsy is indicated to reach the diagnosis earlier for a better prognosis.

## Conclusions

PTC is the most frequent thyroid nodule and always should be included in the differential diagnosis when patients present with lateral neck cystic mass. Therefore, an excisional biopsy should be considered even if the FNA result was benign.
